# The pressing shortage of buprenorphine prescribers and the pending role of telemedicine

**DOI:** 10.1186/1940-0640-10-S1-A40

**Published:** 2015-02-20

**Authors:** Todd Molfenter

**Affiliations:** 1 The Center for Health Enhancement Systems Studies, University of Wisconsin-Madison, Madison, WI, 53706, USA

## Background

The Drug Addiction Treatment Act of 2000 created an opportunity for primary care physicians and addiction treatment agencies to integrate, as primary care physicians became able to prescribe opiate treatment medications from their practices. Fourteen years later, a National Institute on Drug Abuse evidence-based practice implementation trial in Ohio is finding that physician capacity is becoming a primary barrier to use of buprenorphine. This presentation will document: a) the degree of the physician capacity barrier for specialty addiction treatment providers wanting to expand their buprenorphine programs; b) strategies being considered to overcome this barrier, including telemedicine; and c) what workflow challenges implementation of telemedicine can expect (based on the experience of a Veterans Administration [VA] telemedicine study and qualitative analysis of telemedicine implementation in several facilities).

## Methods

The mixed-methods approach documents physician capacity limitations and to what degree telemedicine is being considered to remedy physician capacity shortfalls. Data collection includes written surveys from 47 treatment centers; qualitative interviews with 39 treatment centers; and patient simulations of VA telemedicine programs. For the data analysis, summary statistics are provided, including characteristics of organizational participants and buprenorphine prescribing patterns. The qualitative inductive analysis is designed to identify contextual and process factors that affect telemedicine implementation.

## Results

Fifty percent (sample = 42) of Ohio treatment providers report lack of access to buprenorphine-prescribing physicians as a barrier to implementation and expanded use of buprenorphine. Thirty-eight percent of those identifying this barrier consider telemedicine as an option to access physician prescribers. Barriers to telemedicine implementation are technology incompatibility; inability for telemedicine sites and specialty treatment providers to agree on dosing protocols (including diversion prevention expectations); and workflow interruptions that occur due to patient and clinical information not being effectively transferred between telemedicine sites and community treatment providers. Organizational strategies to overcome lack of physician capacity and telemedicine implementation challenges are discussed.

## Conclusion and implications

The lack of physician-prescribing capacity for buprenorphine is preventing this evidence-based practice from achieving higher penetration rates among specialty treatment providers. Telemedicine provides one solution to re-allocate the distribution of this scarce resource. However, there will also be challenges in implementing telemedicine that need to be understood, and evidence-based strategies need to be developed to overcome these challenges. Successful use of telemedicine may ultimately lead to greater integration between primary care and specialty addiction treatment.

## Trial registration

Clinicaltrials.gov NCT01702142

## Grant support

NIDA 5R01DA030431-03

